# From weight loss to weight gain: appetite changes in major depressive disorder as a mirror into brain-environment interactions

**DOI:** 10.3389/fpsyg.2013.00873

**Published:** 2013-11-21

**Authors:** Gregory J. Privitera, Melissa L. Misenheimer, P. Murali Doraiswamy

**Affiliations:** ^1^Department of Psychology, St. Bonaventure UniversityBonaventure, NY, USA; ^2^Department of Psychiatry, Duke Institute for Brain Sciences and the Duke Brain and Society program, Duke University Medical CenterDurham, NC, USA

**Keywords:** dopaminergic pathways, mood, depression, reward system, atypical depression, sociocultural shifts

## A shift in the appetitive symptoms of depression

In recent decades, there has been a profound shift in the appetitive characteristics of depression. For early measures of depression, such as the Hamilton Rating Scale for Depression (HRSD; Hamilton, 1960), the key appetitive characteristic of depression was weight loss. In the 1950s and 60s, reduced appetite was considered a key feature even in mild depression and it was noted that in moderate depression the desire for food may almost totally disappear (Beck, 1967); those suffering with severe depression may almost have to force themselves to eat (Schuyler, 1974; Polivy and Herman, 1976). Zung, another pioneer of depression measurement, found that even the individual designated as the significant “other” associated loss of appetite as symptomatic of depression in the patient (Zung et al., 1974). No assessment for increased appetite, food intake, or weight gain was assessed in the HRSD. In these early reports, people with depression who overate or gained weight were diagnosed with “atypical depression” and were treated differently than those with more “typical” depression characterized by weight loss.

Yet, this understanding of depression has shifted, with weight gain and increased appetite being identified as a “typical” symptom of depression among those with the disorder today (Doraiswamy, 2013), as is also evident as a common symptom for many other disorders involving emotional distress (American Psychological Association, 2013). Indeed, a recent representative US national survey of 43,093 adults found that the prevalence of major depression with atypical features was almost 40% higher than that of depression without atypical features (Blanco et al., 2012). In this survey, factors that predicted atypical depression appear to mirror the growth of obesity and over eating in our society as a whole.

## Early explanations for weight loss in depression

Original explanations for weight loss observed in patients with depression were based on the need for survival. Reduced appetite can protect humans from food borne diseases. However, this response may not be as necessary today, as food has become increasingly safer to eat and medicine has advanced, having led to increased human life expectancy by almost 10 years since 1960 (Organization for Economic Co-operation and Development, 2011). Another explanation for observed weight loss in patients with depression is based on the behavioral shutdown model, which posits that reduced appetite helps conserve energy in a hostile environment. Today, in developed countries, such as America, however, there is an abundant supply of food that potentially negates the need for a “behavioral shutdown.” Indeed, studies have shown that migration from Mexico to the US is associated with increased binge eating patterns and weight gain (Tavernise, 2013). Hence, early explanations for weight loss observed among patients with depression may now be outdated, with a shift to weight gain reflecting advances in medicine, and societal-cultural changes in the last 50+ years potentially leading to natural biological adaptations.

## From weight loss to weight gain: future directions

While this shift from weight loss to weight gain is evident, little research has specifically investigated how to explain it. Many possible directions for future research can and should be pursued. One promising direction explains that as “comfort foods” (those typically high in fat and sugar) become increasingly available in an environment, as it has over the last half century, those with depression will increasingly seek out those foods for comfort, or to feel good (Privitera, 2008). In line with this explanation, another posits that we are hedonic eaters, i.e., we eat to experience pleasure, not simply satiety or to meet energy needs of the body (Privitera, 2008; Doraiswamy, 2013). Consistent with this theory, recent pilot data suggests an association between preference for sweet taste and depression in obese patients (Aguayo et al., 2012), and that the hedonic response to sweet taste is associated with elevated sensitivity to the mood altering effects of sweet-tasting foods (Kampov-Polevoy et al., 2006).

MDD triggers neurobiological changes specific to the social context within which an individual resides, resulting in somatic symptoms that are then diagnosed within a cultural lens. Hence, as culture changes, so also may symptoms of depression. This is consistent with an influential theory that environmental changes may contribute to the biology of depression through chromatin remodeling and epigenetic changes in gene expression within limbic and neural reward circuits (Nestler, 2012). If this influential theory is true, then transgenerational implications of such shifts would be important to study further.

In addition, hedonic eating may be a product of society-culture. In America, for example, where rates of depression are high, societal demands for two-parent working homes, limited time to prepare and cook meals, and increasingly larger portion sizes and food availability since 1960 may all contribute to enhancing the powerful effects of the midbrain dopamine system to influence food choice for higher fat and sugar foods. From an evolutionary context, sugar indicates safety in nature, and fat is the most calorically dense nutrient on Earth. Hence, the midbrain dopamine system evolved to pursue these nutrients without discrimination (Privitera, 2008); the more available those nutrients are in an environment, the more often we predict that such foods would be pursued and eaten. This could be particularly relevant for patients with depression because intake of these “comfort foods” is also associated with corresponding physiological and psychological increases in emotional responsiveness and positive mood (Dubé et al., 2005).

## Food, mood, and potential therapies

Recent studies show promising results that could also lead to potential therapies and treatments for people with depression. Neurobiological evidence shows that neural reward pathways, and dopaminergic pathways in particular, are activated by the visual presentation of high calorie foods (Frank et al., 2010). Behavioral data show supporting evidence that mood is also positively enhanced among participants who view high calorie and sweet-tasting foods (Privitera et al., 2013a) and participants who draw these foods in an art therapy setting (Privitera et al., 2013b). Hence, studies are now showing positive effects on mood using food cues, without having participants actually consume those foods. To date, these studies were conducted with non-depressed samples. However, the baseline measure of mood for participants with depression would be lower, thereby leading to greater sensitivity to observe positive mood changes using similar procedures to enhance mood and treat depression. Studies investigating the effectiveness of such a therapy for people with depression, and the short- and long-term benefits of such a therapy to enhance mood could lead to potential strategies to treat depression using food cues, without a corresponding increase in weight gain and caloric intake since participants can enhance mood by simply looking at these foods, without actually consuming them.

While the potential directions proposed here are certainly preliminary, there is no denying the “weight” of evidence for a shift in the appetitive symptoms that are characteristic of those with depression. It is therefore imperative to explain this shift, and to open new directions of research that can lead to potential strategies and therapies to treat the now “typical” depression associated with *increased* appetite and weight gain among those with depression.
